# Methyl 5-methyl-1-(1*H*-pyrazol-3-yl)-1*H*-1,2,3-triazole-4-carboxyl­ate

**DOI:** 10.1107/S1600536814012380

**Published:** 2014-06-07

**Authors:** Xiao-Guang Bai, Chao Feng

**Affiliations:** a Institute of Medicinal Biotechnology, Chinese Academy of Medical Sciences and Peking Union Medical College, Beijing 100050, People’s Republic of China; b School of Chemistry and Chemical Engineering, Southeast University, Nanjing 210096, People’s Republic of China

## Abstract

The asymmetric unit of the title compound, C_8_H_9_N_5_O_2_, contains two independent mol­ecules (*A* and *B*) in which the dihedral angles between the triazole and pyrazole rings are 4.80 (14) and 8.45 (16)°. In the crystal, mol­ecules are linked by N—H⋯N hydrogen bonds into supra­molecular independent *A* and *B* chains propagating along the *b*-axis direction. The crystal structure also features π–π stacking between the aromatic rings of adjacent chains, the centroid–centroid separations being 3.8001 (15), 3.8078 (17), 3.8190 (14) and 3.8421 (15) Å.

## Related literature   

For applications of 1,2,3-triazole and its derivatives, see: Danoun *et al.* (1998 ▶); Manfredini *et al.* (2000 ▶).
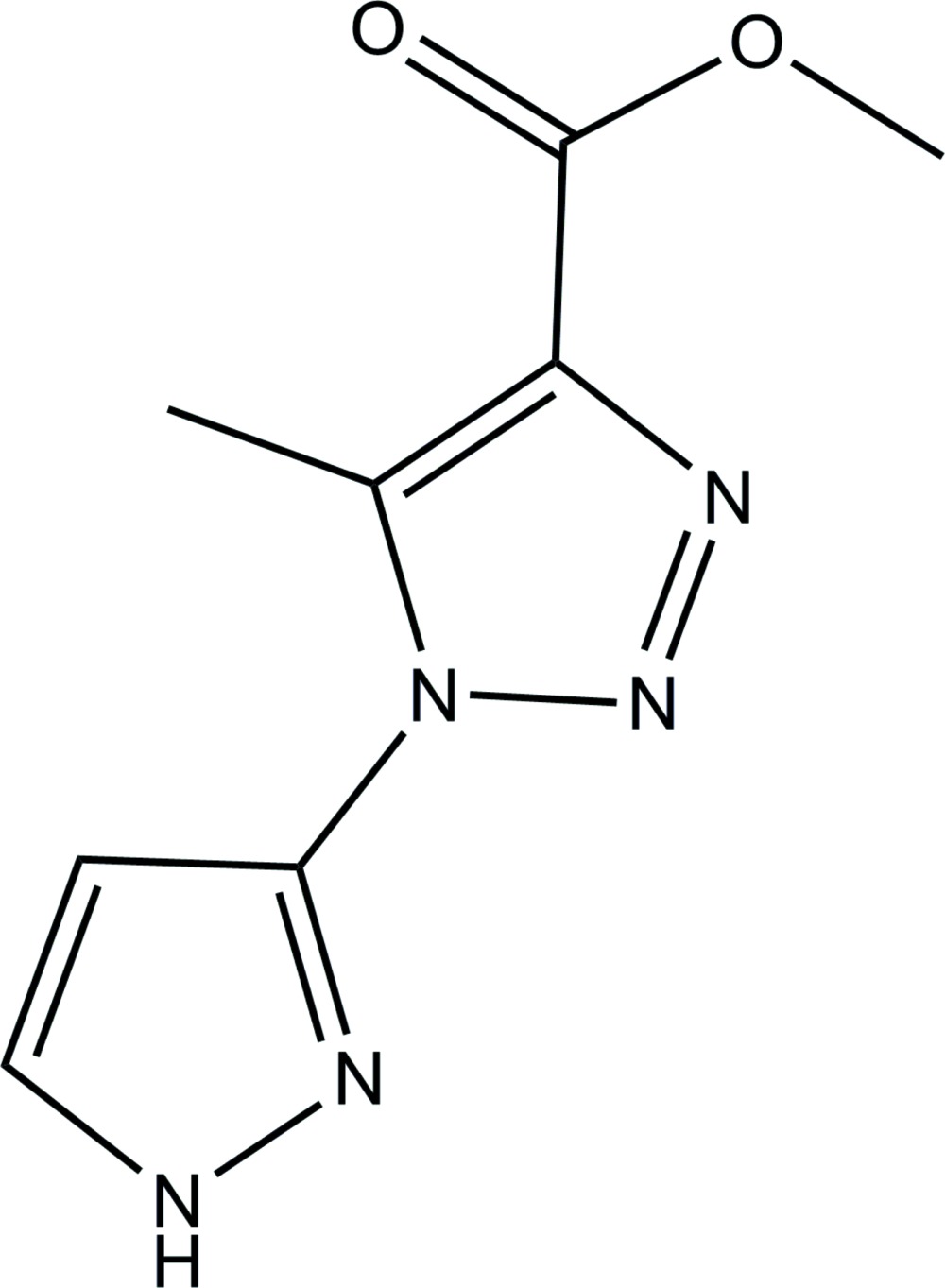


## Experimental   

### 

#### Crystal data  

C_8_H_9_N_5_O_2_
*M* *_r_* = 207.20
Monoclinic, 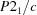

*a* = 15.4576 (6) Å
*b* = 16.0945 (9) Å
*c* = 7.5348 (3) Å
β = 90.079 (4)°
*V* = 1874.52 (15) Å^3^
*Z* = 8
Mo *K*α radiation
μ = 0.11 mm^−1^
*T* = 293 K
0.15 × 0.12 × 0.10 mm

#### Data collection  

Bruker MWPC area-detector diffractometer
5457 measured reflections
3247 independent reflections
2312 reflections with *I* > 2σ(*I*)
*R*_int_ = 0.014

#### Refinement  

*R*[*F* ^2^ > 2σ(*F* ^2^)] = 0.053
*wR*(*F* ^2^) = 0.153
*S* = 1.08
3247 reflections
275 parameters
H-atom parameters constrained
Δρ_max_ = 0.19 e Å^−3^
Δρ_min_ = −0.21 e Å^−3^

### 

Data collection: *FRAMBO* (Bruker, 2004 ▶); cell refinement: *SAINT* (Bruker, 2004 ▶); data reduction: *SAINT*; program(s) used to solve structure: *SHELXTL* (Sheldrick, 2008 ▶); program(s) used to refine structure: *SHELXTL*; molecular graphics: *SHELXTL*; software used to prepare material for publication: *SHELXTL*.

## Supplementary Material

Crystal structure: contains datablock(s) I, New_Global_Publ_Block. DOI: 10.1107/S1600536814012380/xu5793sup1.cif

Structure factors: contains datablock(s) I. DOI: 10.1107/S1600536814012380/xu5793Isup2.hkl

CCDC reference: 1005571

Additional supporting information:  crystallographic information; 3D view; checkCIF report

## Figures and Tables

**Table 1:** Hydrogen-bond geometry (Å, °)

*D*—H⋯*A*	*D*—H	H⋯*A*	*D*⋯*A*	*D*—H⋯*A*
N4—H4*N*⋯N3^i^	0.86	2.17	3.022 (3)	170
N9—H9*N*⋯N8^ii^	0.86	2.20	3.044 (3)	169

Symmetry codes: (i) 
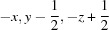
; (ii) 
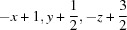
.

**Fig. 1.**
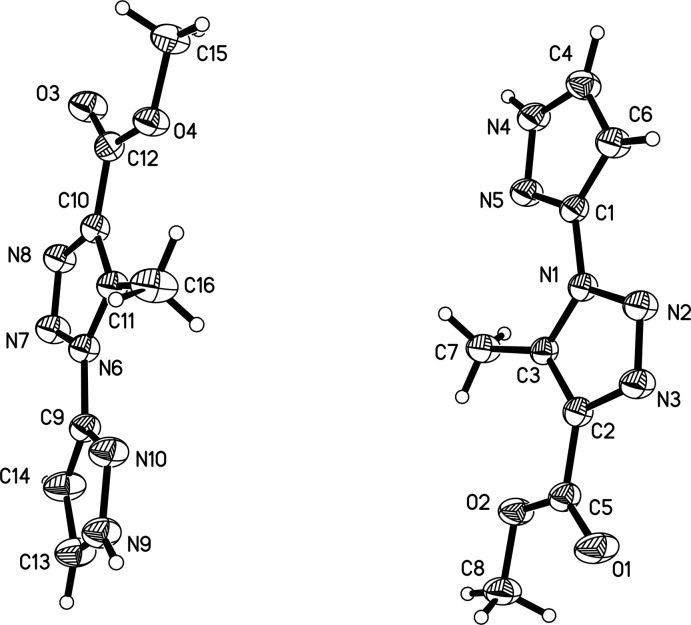
The molecular structure of the title compound with displacement ellipsoids drawn at the 30% probability level.

**Fig. 2.**
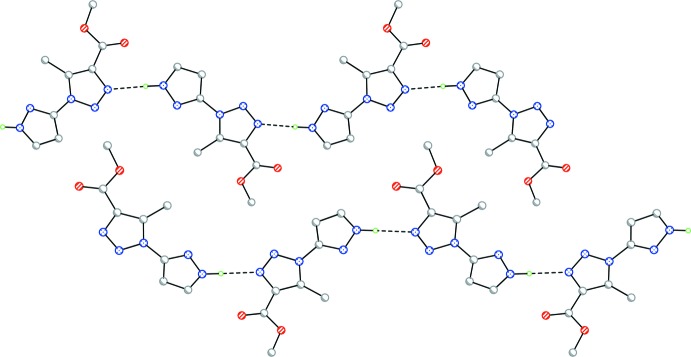
View of the one-dimensional chains of the title compound extending along the b axis. All the hydrogen atoms except those involved in hydrogen bonding have been omitted for clarity. Hydrogen bonds are shown as dashed lines.

## supplementary crystallographic information

### S1. Comment

1,2,3-Triazole and its derivatives had attracted considerable attention for the past few decades due to their chemotherapeutical value. Many 1,2,3-triazoles are found to be potent antimicrobial and antiviral agents. Some of them have exhibited antiproliferative and anticancer activities (Danoun *et al.*, 1998). Some 1,2,3-triazoles are used as DNA cleaving agents (Manfredini *et al.*, 2000) and potassium channel activators. Prompted by the chemotherapeutic importance of 1,2,3-triazoles and its derivatives, we have synthesized the title compound and report its crystal structure herein.

The title compound, contains two crystallographically independent molecules and bond lengths and angles are in the normal range(Fig. 1). The dihedral angle between the triazole and pyrazole rings is 4.80 (14)° and 8.45 (16)° respectively. The crystal structure is stabilized by N–H···N hydrogen bonds linking molecules into one-dimensional chains running parallel to the *b* axis (Fig. 2). The structure is further stabilized by π···π stacking interactions, with centroid-to-centroid separations of 3.8001 (15)–3.8421 (15) Å.

### S2. Experimental

3-Azido-1*H*-pyrazole (20 mmol) was treated with ethyl acetoacetate (24 mmol) in methanol (75 ml) and the mixture was cooled to 273 K. Sodium methoxide (24 mmol) was added to the above mixture and stirred at ambient temperature for 24 h. After completion of the reaction, the mixture was poured on to ice cold water. The precipitated solid was filtered, washed with water and recrystallized from methanol, then 5-methyl-1-(1*H*-pyrazol-3-yl)-1*H*-1,2,3-triazole-4- carboxylic acid were obtained. A mixture of 5-methyl-1-(1*H*-pyrazol-3-yl)-1*H*-1,2,3-triazole-4- carboxylic acid (0.1 mmol) and Et_3_N(0.2 mmol) in methanol (15 ml) was stirred at room temperature until the starting material disappeared. The resulting mixture was filtered and let the filtrate still for 24 h, colorless needle-like crystals were obtained.

### S3. Refinement

H-atoms were placed in calculated positions and refined constrained to ride on their parent atoms, with C—H = 0.93—0.96 Å and N—H = 0.86 Å, *U*_iso_(H) = 1.5*U*_eq_(C) for methyl H atoms and 1.2U_eq_(C,N) for the others.

### Crystal data

**Table:**

C_8_H_9_N_5_O_2_	*F*(000) = 864
*M**_r_* = 207.20	*D*_x_ = 1.468 Mg m^−^^3^
Monoclinic, *P*2_1_/*c*	Mo *K*α radiation, λ = 0.71073 Å
Hall symbol: -P 2ybc	Cell parameters from 1802 reflections
*a* = 15.4576 (6) Å	θ = 3.2–28.8°
*b* = 16.0945 (9) Å	µ = 0.11 mm^−^^1^
*c* = 7.5348 (3) Å	*T* = 293 K
β = 90.079 (4)°	Needle, colorless
*V* = 1874.52 (15) Å^3^	0.15 × 0.12 × 0.10 mm
*Z* = 8	

### Data collection

**Table:**

Bruker MWPC area-detector diffractometer	2312 reflections with *I* > 2σ(*I*)
Radiation source: fine-focus sealed tube	*R*_int_ = 0.014
Graphite monochromator	θ_max_ = 25.2°, θ_min_ = 2.9°
Detector resolution: 0 pixels mm^-1^	*h* = −18→18
phi and ω scans	*k* = −13→19
5457 measured reflections	*l* = −9→8
3247 independent reflections	

### Refinement

**Table:**

Refinement on *F*^2^	Primary atom site location: structure-invariant direct methods
Least-squares matrix: full	Secondary atom site location: difference Fourier map
*R*[*F*^2^ > 2σ(*F*^2^)] = 0.053	Hydrogen site location: inferred from neighbouring sites
*wR*(*F*^2^) = 0.153	H-atom parameters constrained
*S* = 1.08	*w* = 1/[σ^2^(*F*_o_^2^) + (0.0605*P*)^2^ + 0.760*P*] where *P* = (*F*_o_^2^ + 2*F*_c_^2^)/3
3247 reflections	(Δ/σ)_max_ = 0.001
275 parameters	Δρ_max_ = 0.19 e Å^−^^3^
0 restraints	Δρ_min_ = −0.21 e Å^−^^3^

### Special details

**Table:**

Geometry. All esds (except the esd in the dihedral angle between two l.s. planes) are estimated using the full covariance matrix. The cell esds are taken into account individually in the estimation of esds in distances, angles and torsion angles; correlations between esds in cell parameters are only used when they are defined by crystal symmetry. An approximate (isotropic) treatment of cell esds is used for estimating esds involving l.s. planes.
Refinement. Refinement of *F*^2^ against ALL reflections. The weighted *R*-factor *wR* and goodness of fit *S* are based on *F*^2^, conventional *R*-factors *R* are based on *F*, with *F* set to zero for negative *F*^2^. The threshold expression of *F*^2^ > σ(*F*^2^) is used only for calculating *R*-factors(gt) *etc*. and is not relevant to the choice of reflections for refinement. *R*-factors based on *F*^2^ are statistically about twice as large as those based on *F*, and *R*- factors based on ALL data will be even larger.

### Fractional atomic coordinates and isotropic or equivalent isotropic displacement parameters (Å^2^)

**Table:**

	*x*	*y*	*z*	*U*_iso_*/*U*_eq_	
N1	0.02649 (13)	0.54949 (11)	0.2168 (3)	0.0420 (5)	
N2	−0.00324 (14)	0.62920 (13)	0.2245 (3)	0.0541 (6)	
N3	0.05838 (14)	0.67707 (13)	0.1660 (3)	0.0543 (6)	
N4	−0.07242 (14)	0.36437 (14)	0.3301 (3)	0.0537 (6)	
H4N	−0.0746	0.3111	0.3380	0.064*	
N5	−0.00456 (14)	0.40638 (13)	0.2603 (3)	0.0530 (6)	
N6	0.46882 (13)	0.32571 (12)	0.7645 (3)	0.0439 (5)	
N7	0.49447 (14)	0.24550 (13)	0.7423 (3)	0.0526 (6)	
N8	0.43181 (14)	0.19849 (13)	0.7993 (3)	0.0497 (6)	
N9	0.57547 (15)	0.50986 (14)	0.6752 (3)	0.0583 (6)	
H9N	0.5810	0.5630	0.6799	0.070*	
N10	0.50816 (15)	0.46815 (13)	0.7472 (3)	0.0573 (6)	
O1	0.21506 (14)	0.74272 (12)	0.0353 (3)	0.0738 (7)	
O2	0.26259 (12)	0.61387 (11)	−0.0124 (3)	0.0578 (5)	
O3	0.27863 (13)	0.13456 (11)	0.9465 (3)	0.0632 (6)	
O4	0.22679 (11)	0.26408 (11)	0.9707 (3)	0.0539 (5)	
C1	−0.02877 (16)	0.48505 (14)	0.2760 (3)	0.0418 (6)	
C2	0.12793 (16)	0.62903 (15)	0.1203 (3)	0.0437 (6)	
C3	0.10864 (15)	0.54705 (14)	0.1526 (3)	0.0404 (6)	
C4	−0.13523 (18)	0.41433 (17)	0.3852 (4)	0.0551 (7)	
H4	−0.1872	0.3978	0.4365	0.066*	
C5	0.20503 (17)	0.66914 (16)	0.0457 (4)	0.0480 (6)	
C6	−0.11000 (18)	0.49401 (16)	0.3534 (4)	0.0543 (7)	
H6	−0.1399	0.5428	0.3775	0.065*	
C7	0.15978 (18)	0.46989 (16)	0.1315 (4)	0.0597 (8)	
H7A	0.1607	0.4401	0.2419	0.090*	
H7B	0.2179	0.4837	0.0976	0.090*	
H7C	0.1339	0.4358	0.0414	0.090*	
C8	0.33943 (19)	0.64841 (19)	−0.0931 (5)	0.0678 (9)	
H8A	0.3235	0.6797	−0.1966	0.102*	
H8B	0.3778	0.6042	−0.1265	0.102*	
H8C	0.3680	0.6843	−0.0098	0.102*	
C9	0.52627 (17)	0.38975 (15)	0.7090 (3)	0.0450 (6)	
C10	0.36599 (16)	0.24783 (15)	0.8580 (3)	0.0414 (6)	
C11	0.38853 (16)	0.32974 (14)	0.8365 (3)	0.0429 (6)	
C12	0.28750 (17)	0.20845 (16)	0.9289 (3)	0.0449 (6)	
C13	0.6320 (2)	0.46024 (18)	0.5970 (4)	0.0648 (8)	
H13	0.6826	0.4764	0.5399	0.078*	
C14	0.6020 (2)	0.38078 (18)	0.6156 (4)	0.0655 (8)	
H14	0.6271	0.3319	0.5746	0.079*	
C15	0.14627 (18)	0.23064 (18)	1.0380 (4)	0.0608 (8)	
H15A	0.1207	0.1951	0.9500	0.091*	
H15B	0.1074	0.2754	1.0646	0.091*	
H15C	0.1574	0.1992	1.1438	0.091*	
C16	0.3426 (2)	0.40823 (17)	0.8776 (5)	0.0707 (9)	
H16A	0.3720	0.4365	0.9724	0.106*	
H16B	0.2844	0.3960	0.9130	0.106*	
H16C	0.3418	0.4430	0.7742	0.106*	

### Atomic displacement parameters (Å^2^)

**Table:**

	*U*^11^	*U*^22^	*U*^33^	*U*^12^	*U*^13^	*U*^23^
N1	0.0437 (11)	0.0246 (11)	0.0577 (13)	0.0050 (9)	0.0077 (10)	−0.0008 (9)
N2	0.0512 (13)	0.0265 (11)	0.0846 (16)	0.0056 (10)	0.0190 (12)	0.0008 (11)
N3	0.0536 (13)	0.0297 (12)	0.0797 (16)	0.0022 (10)	0.0166 (12)	0.0000 (11)
N4	0.0569 (14)	0.0294 (12)	0.0749 (15)	−0.0065 (11)	0.0073 (12)	0.0048 (11)
N5	0.0510 (12)	0.0304 (12)	0.0775 (16)	−0.0007 (10)	0.0115 (11)	0.0028 (11)
N6	0.0485 (12)	0.0262 (11)	0.0569 (13)	0.0037 (9)	0.0037 (10)	−0.0012 (9)
N7	0.0490 (13)	0.0283 (12)	0.0806 (16)	0.0044 (10)	0.0112 (11)	−0.0007 (11)
N8	0.0476 (12)	0.0287 (11)	0.0729 (15)	0.0046 (10)	0.0064 (11)	0.0003 (10)
N9	0.0654 (15)	0.0321 (12)	0.0774 (16)	−0.0088 (11)	0.0060 (13)	0.0001 (11)
N10	0.0628 (14)	0.0295 (12)	0.0797 (16)	−0.0052 (11)	0.0098 (13)	−0.0016 (11)
O1	0.0751 (14)	0.0326 (11)	0.1139 (18)	−0.0044 (10)	0.0299 (13)	0.0005 (11)
O2	0.0488 (11)	0.0380 (11)	0.0865 (14)	−0.0008 (9)	0.0176 (10)	0.0030 (9)
O3	0.0636 (12)	0.0309 (10)	0.0953 (15)	−0.0041 (9)	0.0127 (11)	0.0010 (10)
O4	0.0461 (10)	0.0376 (10)	0.0780 (13)	0.0013 (8)	0.0139 (9)	0.0032 (9)
C1	0.0461 (14)	0.0291 (13)	0.0502 (14)	−0.0016 (11)	0.0014 (11)	−0.0004 (11)
C2	0.0452 (14)	0.0308 (13)	0.0552 (15)	0.0034 (11)	0.0046 (12)	−0.0029 (11)
C3	0.0443 (13)	0.0293 (13)	0.0477 (14)	0.0044 (11)	0.0051 (11)	0.0002 (11)
C4	0.0481 (15)	0.0448 (16)	0.0724 (19)	−0.0039 (13)	0.0119 (14)	−0.0014 (14)
C5	0.0517 (15)	0.0316 (14)	0.0608 (16)	0.0005 (12)	0.0060 (13)	−0.0007 (12)
C6	0.0529 (15)	0.0351 (15)	0.0750 (19)	0.0016 (13)	0.0137 (14)	−0.0020 (13)
C7	0.0509 (15)	0.0355 (15)	0.093 (2)	0.0072 (13)	0.0223 (15)	0.0047 (14)
C8	0.0522 (16)	0.0539 (19)	0.097 (2)	−0.0017 (15)	0.0252 (16)	0.0082 (17)
C9	0.0488 (15)	0.0295 (14)	0.0569 (16)	−0.0011 (11)	0.0011 (12)	0.0016 (11)
C10	0.0449 (13)	0.0299 (13)	0.0496 (15)	0.0036 (11)	0.0013 (11)	−0.0025 (11)
C11	0.0466 (14)	0.0277 (13)	0.0543 (15)	0.0027 (11)	0.0050 (11)	−0.0023 (11)
C12	0.0494 (15)	0.0340 (14)	0.0511 (15)	0.0001 (12)	−0.0010 (12)	0.0000 (12)
C13	0.0654 (18)	0.0450 (18)	0.084 (2)	−0.0062 (15)	0.0177 (16)	0.0031 (15)
C14	0.0701 (19)	0.0386 (16)	0.088 (2)	0.0007 (14)	0.0264 (17)	−0.0005 (15)
C15	0.0483 (16)	0.0502 (18)	0.084 (2)	−0.0022 (13)	0.0157 (15)	0.0002 (15)
C16	0.0689 (19)	0.0316 (15)	0.112 (3)	0.0045 (14)	0.0308 (18)	−0.0092 (16)

### Geometric parameters (Å, º)

**Table:**

N1—C3	1.360 (3)	C2—C3	1.374 (3)
N1—N2	1.364 (3)	C2—C5	1.468 (4)
N1—C1	1.416 (3)	C3—C7	1.481 (3)
N2—N3	1.302 (3)	C4—C6	1.362 (4)
N3—C2	1.369 (3)	C4—H4	0.9300
N4—C4	1.328 (3)	C6—H6	0.9300
N4—N5	1.355 (3)	C7—H7A	0.9600
N4—H4N	0.8600	C7—H7B	0.9600
N5—C1	1.326 (3)	C7—H7C	0.9600
N6—C11	1.357 (3)	C8—H8A	0.9600
N6—N7	1.361 (3)	C8—H8B	0.9600
N6—C9	1.424 (3)	C8—H8C	0.9600
N7—N8	1.302 (3)	C9—C14	1.375 (4)
N8—C10	1.365 (3)	C10—C11	1.373 (3)
N9—C13	1.323 (4)	C10—C12	1.470 (4)
N9—N10	1.352 (3)	C11—C16	1.482 (3)
N9—H9N	0.8600	C13—C14	1.368 (4)
N10—C9	1.324 (3)	C13—H13	0.9300
O1—C5	1.197 (3)	C14—H14	0.9300
O2—C5	1.333 (3)	C15—H15A	0.9600
O2—C8	1.446 (3)	C15—H15B	0.9600
O3—C12	1.204 (3)	C15—H15C	0.9600
O4—C12	1.335 (3)	C16—H16A	0.9600
O4—C15	1.448 (3)	C16—H16B	0.9600
C1—C6	1.392 (4)	C16—H16C	0.9600
			
C3—N1—N2	110.93 (19)	H7A—C7—H7B	109.5
C3—N1—C1	130.9 (2)	C3—C7—H7C	109.5
N2—N1—C1	118.16 (19)	H7A—C7—H7C	109.5
N3—N2—N1	107.18 (19)	H7B—C7—H7C	109.5
N2—N3—C2	109.0 (2)	O2—C8—H8A	109.5
C4—N4—N5	112.7 (2)	O2—C8—H8B	109.5
C4—N4—H4N	123.6	H8A—C8—H8B	109.5
N5—N4—H4N	123.6	O2—C8—H8C	109.5
C1—N5—N4	102.9 (2)	H8A—C8—H8C	109.5
C11—N6—N7	111.2 (2)	H8B—C8—H8C	109.5
C11—N6—C9	130.9 (2)	N10—C9—C14	113.1 (2)
N7—N6—C9	118.0 (2)	N10—C9—N6	119.6 (2)
N8—N7—N6	107.08 (19)	C14—C9—N6	127.3 (2)
N7—N8—C10	108.9 (2)	N8—C10—C11	109.3 (2)
C13—N9—N10	112.8 (2)	N8—C10—C12	118.9 (2)
C13—N9—H9N	123.6	C11—C10—C12	131.8 (2)
N10—N9—H9N	123.6	N6—C11—C10	103.5 (2)
C9—N10—N9	102.9 (2)	N6—C11—C16	124.3 (2)
C5—O2—C8	115.5 (2)	C10—C11—C16	132.2 (2)
C12—O4—C15	116.0 (2)	O3—C12—O4	123.8 (2)
N5—C1—C6	113.0 (2)	O3—C12—C10	124.1 (2)
N5—C1—N1	120.0 (2)	O4—C12—C10	112.2 (2)
C6—C1—N1	126.9 (2)	N9—C13—C14	107.1 (3)
N3—C2—C3	109.1 (2)	N9—C13—H13	126.4
N3—C2—C5	119.1 (2)	C14—C13—H13	126.4
C3—C2—C5	131.8 (2)	C13—C14—C9	104.1 (3)
N1—C3—C2	103.8 (2)	C13—C14—H14	128.0
N1—C3—C7	124.1 (2)	C9—C14—H14	128.0
C2—C3—C7	132.1 (2)	O4—C15—H15A	109.5
N4—C4—C6	107.8 (2)	O4—C15—H15B	109.5
N4—C4—H4	126.1	H15A—C15—H15B	109.5
C6—C4—H4	126.1	O4—C15—H15C	109.5
O1—C5—O2	123.5 (3)	H15A—C15—H15C	109.5
O1—C5—C2	124.4 (2)	H15B—C15—H15C	109.5
O2—C5—C2	112.0 (2)	C11—C16—H16A	109.5
C4—C6—C1	103.6 (2)	C11—C16—H16B	109.5
C4—C6—H6	128.2	H16A—C16—H16B	109.5
C1—C6—H6	128.2	C11—C16—H16C	109.5
C3—C7—H7A	109.5	H16A—C16—H16C	109.5
C3—C7—H7B	109.5	H16B—C16—H16C	109.5

### Hydrogen-bond geometry (Å, º)

**Table:**

*D*—H···*A*	*D*—H	H···*A*	*D*···*A*	*D*—H···*A*
N4—H4*N*···N3^i^	0.86	2.17	3.022 (3)	170
N9—H9*N*···N8^ii^	0.86	2.20	3.044 (3)	169

Symmetry codes: (i) −*x*, *y*−1/2, −*z*+1/2; (ii) −*x*+1, *y*+1/2, −*z*+3/2.
